# Human Peripheral Blood Mononuclear Cells Exhibit Heterogeneous CD52 Expression Levels and Show Differential Sensitivity to Alemtuzumab Mediated Cytolysis

**DOI:** 10.1371/journal.pone.0039416

**Published:** 2012-06-25

**Authors:** Sambasiva P. Rao, Jose Sancho, Juanita Campos-Rivera, Paula M. Boutin, Peter B. Severy, Timothy Weeden, Srinivas Shankara, Bruce L. Roberts, Johanne M. Kaplan

**Affiliations:** 1 Neuroimmunology Research, Genzyme, A Sanofi Company, Framingham, Massachusetts, United States of America; 2 Immune Mediated Disease Biology Research, Genzyme, A Sanofi Company, Framingham, Massachusetts, United States of America; Centre de Recherche Public de la Santé (CRP-Santé), Luxembourg

## Abstract

Alemtuzumab is a monoclonal antibody that targets cell surface CD52 and is effective in depleting lymphocytes by cytolytic effects in vivo. Although the cytolytic effects of alemtuzumab are dependent on the density of CD52 antigen on cells, there is scant information regarding the expression levels of CD52 on different cell types. In this study, CD52 expression was assessed on phenotypically distinct subsets of lymphoid and myeloid cells in peripheral blood mononuclear cells (PBMCs) from normal donors. Results demonstrate that subsets of PBMCs express differing levels of CD52. Quantitative analysis showed that memory B cells and myeloid dendritic cells (mDCs) display the highest number while natural killer (NK) cells, plasmacytoid dendritic cells (pDCs) and basophils have the lowest number of CD52 molecules per cell amongst lymphoid and myeloid cell populations respectively. Results of complement dependent cytolysis (CDC) studies indicated that alemtuzumab mediated profound cytolytic effects on B and T cells with minimal effect on NK cells, basophils and pDCs, correlating with the density of CD52 on these cells. Interestingly, despite high CD52 levels, mDCs and monocytes were less susceptible to alemtuzumab-mediated CDC indicating that antigen density alone does not define susceptibility. Additional studies indicated that higher expression levels of complement inhibitory proteins (CIPs) on these cells partially contributes to their resistance to alemtuzumab mediated CDC. These results indicate that alemtuzumab is most effective in depleting cells of the adaptive immune system while leaving innate immune cells relatively intact.

## Introduction

CD52 is a cell surface glycoprotein consisting of a short 12 aa peptide with a C terminal GPI anchor. It is present on human chromosome1 [1] and is known to have two alleles that differ in two bases coding for amino acids at C-terminal side of the GPI attachment region. The two alleles are thought to code for identical mature antigens and individuals of different genotypes do not exhibit phenotypic differences [2]. CD52 is expressed on lymphocytes, monocytes, eosinophils and in the male reproductive tract on epithelial cells of the epididymis and seminal vesicle. The CD52 antigen is secreted into seminal plasma where it is taken up by mature sperm [2], [3].

Alemtuzumab is a humanized monoclonal antibody to human CD52, genetically engineered by grafting rat complementarity determining regions (CDRs) into human framework regions fused to human IgG1 [4]. It binds to the C-terminal part of the peptide to an epitope that includes part of the GPI anchor [5]. Alemtuzumab has been approved for the treatment of patients with advanced chronic lymphocytic leukemia (CLL) [6], [7], [8]. This antibody has also been utilized in the treatment of a wide range of diseases including rheumatoid arthritis [9], [10], [11], non-Hodgkin’s lymphoma [12], [13] and T- cell lymphoma [14], [15]. In recent phase 2 (CAMMS223) clinical studies, alemtuzumab showed efficacy in the treatment of relapsing-remitting multiple sclerosis [16]. Alemtuzumab induces potent cytolysis of CD52 expressing lymphocytes. Although the predominant mechanism of lysis is not certain, antibody dependent cellular cytotolysis and complement dependent cytolysis are presumed to be important [17], [18], [19], [20]. In addition, caspase-8 dependent and independent apoptosis have also been identified as other potential mechanisms of cytolytic action by alemtuzumab on cell lines and CLL cells [21], [22], [23].

Although alemtuzumab has potent cytolytic effects on mature lymphocytes, hematopoietic stem cells (HSCs) and some myeloid derived cells were found to be less sensitive to alemtuzumab mediated depletion [24], [25], [26]. This difference in responsiveness to cytolytic effects of alemtuzumab has been attributed to the relatively lower levels of CD52 expression [24], [25], [26], [27]. These studies highlight the importance of the levels or number of CD52 antigenic determinants on cells to which alemtuzumab can bind which is critical for cytolytic effects, especially complement dependent cytolysis. In this regard, there is scant information regarding the absolute numbers of CD52 antigenic determinants for alemtuzumab on various subsets of PBMC populations and available information is limited to total B and T cells [14], [24], [27], [28]. The cell surface expression and the quantitative levels of CD52 on various lymphocyte and myeloid cell subsets in human blood leukocytes are not known and information pertaining to the correlation between the density of CD52 molecules and cytolytic effects of alemtuzumab on phenotypically distinct subsets is lacking.

In this study, we sought to investigate the qualitative expression and quantitative levels of CD52 antigen density on phenotypically distinct subsets of lymphocyte and myeloid cell populations in peripheral blood mononuclear cells (PBMCs) from normal human donors. In addition, we tested the complement mediated cytolytic effects mediated by alemtuzumab on human PBMC subsets to investigate the correlation between the amount of cytolysis and the quantitative levels of CD52. While cytolytic effects correlated with antigen density on a majority of PBMC subsets, CD52 antigen density did not correlate with the degree of cytolysis for some myeloid cell populations and our studies indicated that they expressed higher levels of complement inhibitory proteins. These results provide new insights into differential cytolytic effects of alemtuzumab on immune cells.

## Materials and Methods

### Donors and Blood Collection

The donors comprised 22 normal individuals from whom fresh blood was obtained after informed consent by drawing 10 ml of venous blood into tubes containing potassium EDTA.

### Antibodies

The following fluorochrome conjugated antibodies were used for flow cytometric analysis: anti CD3-FITC, anti CD27-PE or APC, anti CD46-PE, anti CD55-PE, anti CD59-PE anti CD62L-PE Cy5, anti CD56-PE Cy7, anti CD16-APC Cy7, anti-CD11c-PE Cy5, anti-CD123-PE, (BD Biosciences, San Diego, CA), anti CD3-efluor 650, anti-CD11c- Alexa 700, anti CD123-PE Cy7, anti-HLA-DR-efluor 605, anti-CD19-efluor-450 (ebiosciences, San Diego, CA), anti CD54RA-ECD, anti HLA-DR-ECD (Beckman coulter), anti CD19-Pacific Blue, anti-CD3 pacific blue, anti CD4-APC Cy5.5 and anti-CD8 pacific orange, anti-CD14 pacific orange, (Invitrogen, CA), anti BDCA2-APC (Miltenyi Biotec, Auburn, CA), alemtuzumab-FITC (Genzyme Corporation). Purified anti CD55 - clone BRC 216 (abcam, Cambridge, MA) and purified anti CD59 - clone BRIC 229 (IBGRL Research products, Bristol, UK) were used as neutralizing antibodies in some functional experiments.

### Fluorochrome Labelling of Alemtuzumab

A clinical lot of alemtuzumab was buffer exchanged to a concentration of 10 mg/ml in 0.1 mM NaH_2_CO_3_ pH = 8.0 using a 10 K cutoff spin column (Millipore, Billerica, MA). 5′Fluorescein isothiocyanate (Invitrogen, CA) was dissolved at 10 mg/ml in DMSO and added at a 15 fold molar excess to the antibody. The reaction was carried out at room temp for 2 hrs protected from light. Labeled antibody was gel purified using Zebra-spin column (Thermo Scientific, Rockford, IL) that had been equilibrated with PBS pH = 7.2. Concentration and degree of labeling (moles of dye/mole of protein) were determined using UV absorbance at 280 nm and 494 nm.

### Mononuclear Cell Separation

Blood obtained from normal donors was processed for separation of mononuclear cells. Human peripheral blood was diluted 1∶1 with sterile phosphate-buffered saline (PBS), layered over Ficoll-Hypaque (GE life sciences, Uppsala, Sweden) and centrifuged at 1500 rpm for 30 min at room temperature. The interphase layer of PBMCs was drawn out and the cells were washed in PBS containing 5% fetal bovine serum (FACS buffer). Cells were resuspended in cold FACS buffer and were separated from debris by passing them through a 40 micron cell strainer.

### Flow Cytometry and Cell Sorting

For qualitative analysis of CD52 expression, a lymphocyte cocktail containing pretitered dilutions of fluorescently-labeled antibodies against CD3, CD27, CD45RA, CD62L, CD56, CD19, CD8 or CD4, CD16 and FITC labeled alemtuzumab to identify individual subsets of lymphoid cells and a myeloid cocktail containing pretitered dilutions of fluorescently-labeled antibodies against CD123, HLA-DR, CD11c, CD16, CD14, BDCA-2, CD3 and FITC labeled alemtuzumab to identify myeloid populations were used to perform multicolor flow cytometric analysis. To assess expression of complement inhibitory proteins (CIPs) on lymphoid and myeloid subsets from four of the twenty two normal donors, a cocktail of antibodies to CD27, CD45-RA, CD123, CD19, CD14, CD3, CD8, CD56, CD11c, CD16, CD3 and HLA-DR and PE-conjugated antibodies to either CD46, CD55 or CD59 CIPs were used in separate experiments. Briefly, each cocktail of antibodies was mixed together with 1×10^6^ PBMCs in the wells of a 96-well U-bottom plate and incubated on ice for 30 min. The cells were subsequently washed in FACS buffer and fixed in PBS containing 0.5% paraformaldehyde. One hundred thousand events of the stained cells were acquired on a BD LSR-II cytometer and the data were analyzed using FlowJo 7.2 version Software and expressed as median fluorescence intensity (MFI).

Quantitation of cellular CD52 expression in antibody binding capacity (ABC) units was performed using Quantum Simply Cellular anti-human IgG beads (Bangs Laboratories, Inc; Fisher, IN, USA). The beads are uniform cell-sized microspheres with different calibrated binding capacities of goat anti-human IgG (Fc specific) coated on their surface. There are four coated populations of beads with differing antibody binding capacities (ABC) for human monoclonal antibodies. The beads were labeled with FITC-conjugated alemtuzumab in the same manner as the cells labeled with cocktails of antibodies described above and data were acquired on a BD LSR-II flow cytometer. The median values of fluorescence intensity (MFI) of the beads were converted to ABC units using Quick Cal software to construct a standard calibration curve. Since the PBMCs are labeled and acquired on the same flow cytometer using similar instrument settings as the beads, the standard curve provides a means to convert the median fluorescence intensity values of phenotypically defined subsets into absolute numbers (ABC units) of CD52 molecules on cells.

For cell sorting, freshly isolated PBMCs were stained with antibodies to CD3, CD19, CD56, CD14 and CD11c. Three way sorting was performed on a FACS Aria (Beckton Dickinson, SanDiego, CA) to separate purified populations consisting of CD3+T cell, CD56+ NK cells and CD14+ CD11c+ monocytes after gating out CD19+ B cells in the dump channel. Cytolytic experiments were performed on purified cell populations as described in the next section below.

### Complement-dependent Cytolysis (CDC) Assay

For the CDC assay, 1×10^6^ Ficoll-purified PBMCs were incubated with 10 µg/ml of alemtuzumab or control human IgG in duplicate in a flexible U-bottomed 96 well plate. Human complement (Quidel Corporation, San Diego, CA) was added to the cells at a final concentration of 10% (v/v) and incubated for 3 hrs at 37°C in a humidified atmosphere containing 5% CO_2_. Following CDC, cells were washed with HBSS/4% HSA medium and incubated in FACS buffer with a combination of fluorochrome labeled antibodies to CD27, CD45-RA, CD123, CD19, CD14, CD8, CD56, BDCA-2, CD11c, CD16, CD3 and HLA-DR for 30 min on ice. In some experiments, CDC was performed on purified populations of T cells, NK cells and monocytes in the presence or absence of neutralizing antibodies to CD55 and CD59.The cells were subsequently washed and stained with Annexin-V in 150 µl of binding buffer for 15 min at room temperature. 7-AAD (7.5 µl) was added to the cell suspension and incubated for an additional 10 min after which 25 µl of Count Bright absolute counting beads (Invitrogen, CA) were added to each sample. A minimum of 100,000 events were acquired from each sample on a BD LSR-II flow cytometer and the data were analyzed using FlowJo 7.2 version Software. The number of cell events of each PBMC subset among the cells that survived cytolytic effects was obtained by analyzing equal numbers of cells from control IgG and alemtuzumab treated samples after normalizing the number of cell events by concatenation in Flowjo software. The absolute cell number of each PBMC subset was then calculated using the formula,

# of cell events × assigned bead count of the lot  =  absolute number of cells/100 µl of sample volume

# of bead events.

## Results

### Polychromatic Flow Cytometry (PFC) Reveals Distinct PBMC Subsets

**Figure 1:**
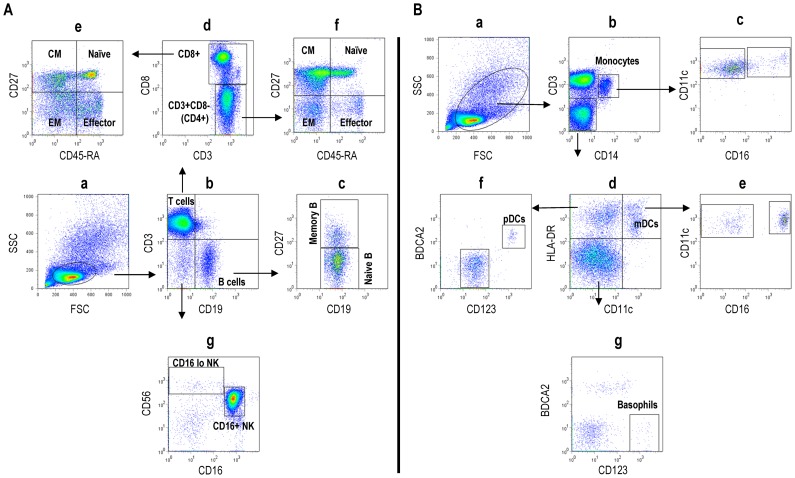
Phenotypic characterization of lymphoid and myeloid subsets. Representative polychromatic flow cytometric analysis of lymphoid (A) and myeloid (B) subsets from PBMCs. Mem B (Memory B-cells), CM (Central memory), EM (Effector memory), NK (Natural killer), mDCs (Myeloid Dendritic cells), pDCs (Plasmacytoid dendritic cells).The phenotype of each PBMC subset is detailed in Table 1.

Multiple subsets of cells representing various stages of differentiation and activation associated with distinct phenotypic and functional characteristics have been defined in human peripheral blood leukocytes [29], [30], [31], [32]. To better define the pattern of expression of CD52 on leukocyte subsets we used PFC to first define and characterize multiple populations of lymphocytes and myeloid cells. Analyzing multiple cell surface markers simultaneously, we first defined phenotypically distinct cell populations corresponding to lymphocyte, myeloid and plasmacytoid cell lineages in PBMCs from 22 normal donors. Figure 1 shows one representative analysis and the phenotype of each individual PBMC subset is provided in Table 1. As shown in Figure 1A, we identified two B cell populations representing naive and memory B cell subsets (Panel c), four subsets each of CD4 and CD8 populations corresponding to naïve, central memory, effector memory and effector T-cells (panels e and f), and two subsets of NK cells based on differential expression of CD16 and CD56 (Panel g). A similar analysis defining populations of myeloid and lymphoid derived plasmacytoid dendritic cells is shown in Figure 1B. Here, we identified two subsets of CD14+ CD11c+ monocytes (Panel c) and two subsets of CD11c+ HLADR+ myeloid DCs based on differential CD16 expression (Panel e). In addition, CD11c negative cells could be separated into plasmacytoid dendritic cells (pDCs) (panel f) and basophils (panel g) based on the expression of HLA-DR, CD123, and BDCA2.

**Table 1:** Cell surface phenotypic characteristics of human PBMC subsets.

Surface Phenotype	Lymphocyte Subset name
CD3− CD19+ CD27−	Naïve − B
CD3− CD19+ CD27+	Memory − B
CD19− CD3+ CD4+ CD8−CD45RA+ CD27+	CD4 − Naïve
CD19− CD3+ CD4+ CD8−CD45RA− CD27+	CD4 − Central Memory
CD19− CD3+ CD4+ CD8−CD45RA− CD27−	CD4 − Effector Memory
CD19− CD3+ CD4+ CD8−CD45RA+ CD27−	CD4 − Effector
CD19− CD3+ CD4− CD8+CD45RA+ CD27+	CD8 − Naïve
CD19− CD3+ CD4− CD8+CD45RA− CD27+	CD8 − Central Memory
CD19− CD3+ CD4− CD8+CD45RA− CD27−	CD8 − Effector Memory
CD19− CD3+ CD4− CD8+CD45RA+ CD27−	CD8 − Effector
CD3−CD19−CD56hi CD16lo	CD16lo NK
CD3−CD19−CD56lo CD16hi	CD16hi NK
**Surface Phenotype**	**Myeloid subset name**
CD3− CD19− HLA−DR+ CD11c+ CD14+ CD16+	CD16+ Monocyte
CD3− CD19− HLA−DR+ CD11c+ CD14+ CD16−	CD16− Monocyte
CD3− CD19− HLA−DR+ CD11c+ CD14− CD16+	CD16+ Myeloid dendritic cell
CD3− CD19− HLA−DR+ CD11c+ CD14− CD16−	CD16− Myeloid dendritic cell
CD3− CD19− HLA−DR+ CD11c− CD14− CD123+ BDCA2+	Plasmacytoid dendritic cell
CD3− CD19− HLA−DR− CD11c− CD14− CD123+	Basophils

### PBMC Subsets Exhibit Differential Expression of CD52

**Figure 2:**
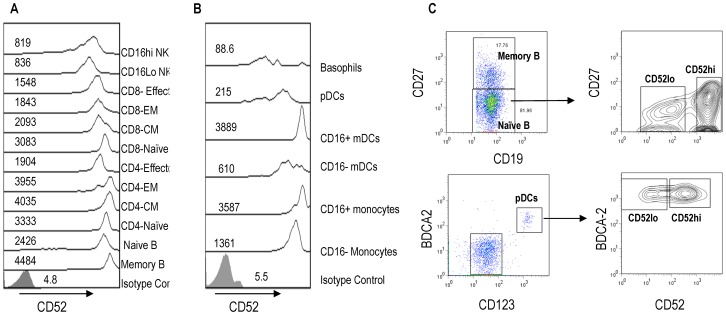
Differential expression of CD52 on human PBMC subsets. Representative histograms of CD52 expression levels on each of the lymphoid (A) and myeloid (B) cell subsets described in figure 1 were analyzed using Flowjo software v7.2. The histograms and the corresponding median fluorescence intensity value of each of the subsets are shown in the panels. **A**: Hierarchy of CD52 levels on lymphoid cells. Mem B > CD4−CM > CD4−EM > CD4−Naïve > CD8−Naïve− > Naïve−B > CD8−CM > CD4−Effector > CD16lo Nk > CD16hi NK. **B**: Hierarchy of CD52 levels on myeloid cells: CD16+ mDC > CD16+ Mono > CD16− Mono > CD16− mDC >pDCs > Basophils. **C**. Representative plots showing heterogenous expression of CD52 on Naïve B cells and pDCs.

Qualitative expression of CD52 on phenotypically defined PBMC subsets as described above was assessed by examining the intensity of staining which corresponds to the ability of alemtuzumab to detect CD52 expression. Representative histograms and median fluorescence intensity (MFI) values from one donor are shown in Figure 2. The results show that there is a significant differential pattern of CD52 expression among the PBMC subsets. The data on lymphocytes (Figure 2A) reveal that while memory B cells exhibit the highest expression followed by subsets of T cells, NK cells show much lower levels of CD52 expression. In nineteen of twenty two donors, naïve B cells showed heterogeneous CD52 expression allowing delineation into CD52hi naïve and CD52lo naïve subsets (Fig 2C and Table S1). Among the myeloid cells (Figure 2B), the CD16+ monocytes and mDCs show higher levels of CD52 expression than their corresponding CD16 low subsets. Basophils exhibit the lowest CD52 expression. Similar to naïve B cells, there was heterogeneous CD52 expression levels on plasmacytoid dendritic cells (pDCs) that allowed separation of these cells into a CD52hi and CD52lo subsets in all the donors examined (Fig 2C and Table S1).

### Quantitative Analysis of CD52 Levels on PBMC Subsets

**Figure 3:**
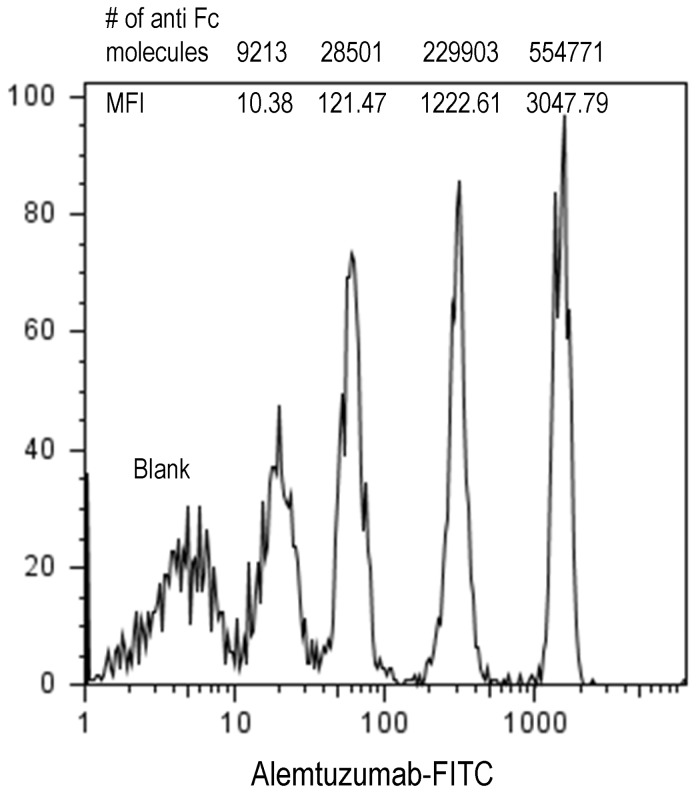
Calibration of Simply Quantum cellular microspheres with alemtuzumab. Uniformly sized microspheres coated with different numbers (shown above each histogram) of anti-human Fc molecules defined as antibody binding capacity (ABC) are incubated with a saturating concentration of FITC-conjugated alemtuzumab (5 µg/ml). The beads were analyzed by flow cytometry on an LSR-II instrument and the median fluorescence intensity (MFI) values (shown next to each histogram) were plotted against the ABC units (shown on top of each histogram) to generate a standard calibration curve (not shown). The cells were labeled with alemtuzumab-FITC in the same manner as the beads and the MFI of CD52 expression on each cell subset was used to quantify absolute CD52 levels in ABC units or number of CD52 molecules per cell using the calibration curve.

**Figure 4:**
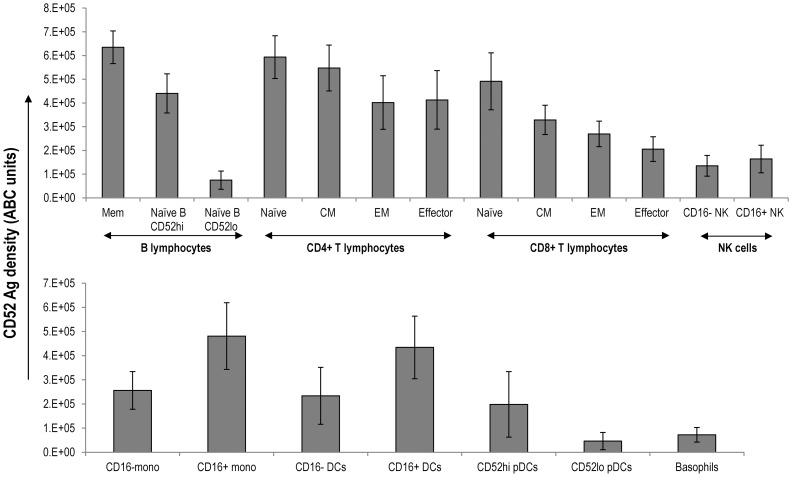
CD52 antigen density on human PBMC subsets. CD52 expression was determined on subsets of freshly isolated PBMCs from twenty two normal donors using the strategy described in Fig 1. The number of alemtuzumab binding units to CD52 antigen was determined from the calibration curve as described in fig 3. The average CD52 antigen density on lymphoid (A) and myeloid (B) subsets of PBMCs is presented with the error bars representing standard deviation.

We next ascertained the absolute numbers of CD52 molecules on each of the individual subsets in PBMCs from all donors in order to evaluate to what extent the variable CD52 expression translates quantitatively. Quantitative flow cytometry was performed using Quantum Simply Cellular beads (see materials and methods) and a calibration curve was generated from the fluorescence intensity values obtained with a saturating concentration of alemtuzumab (Figure 3). PFC was performed on PBMCs and the number of CD52 molecules expressed as ABC units for each of the PBMC subsets were obtained from the standard curve. Mean CD52 levels are summarized in Figure 4 and Table S1 shows CD52 levels on each individual subset from every donor. The hierarchy in quantitative levels of CD52 expression on lymphoid (Figure 4A) and myeloid populations (Figure 4B) mirrors exactly that observed in qualitative expression in all the donors studied. Memory B cells have the highest levels of CD52 expression (Avg  = 634692±68919 ABC units) and the CD16lo CD56 hi NK subset expresses the lowest levels (Avg  = 135418±43632 ABC units). CD52 antigen density on the subsets of CD4 T cells was much higher than on the corresponding subsets of CD8 T cells. The effector CD8 T-cells have lower average levels of CD52 antigen compared to other T cell subsets in the majority of donors (Avg  = 205559±51904 ABC units). Among the myeloid cell populations, CD16+ monocyte (Avg  = 481083±137931 ABC units) and CD16+ DC subsets (Avg  = 434011±129432 ABC units) express the highest CD52 levels. CD16 negative monocytes and mDCs show much lower average levels of CD52 than CD16+ subsets although the average levels were higher to those expressed by CD8 effector cells. Basophils (Avg  = 72308±30230 ABC units) show the lowest CD52 expression levels. This trend of differential CD52 expression pattern was consistent in all the donors.

These results provide the first comprehensive analysis of qualitative and quantitative expression levels of CD52 on human lymphoid and myeloid PBMC subsets. They demonstrate that CD52 expression among PBMC subsets is highly heterogeneous but follows the same hierarchical expression pattern across multiple normal donors examined.

### PBMC Subsets Exhibit Differential Susceptibility to Alemtuzumab Mediated Cytolysis

**Figure 5:**
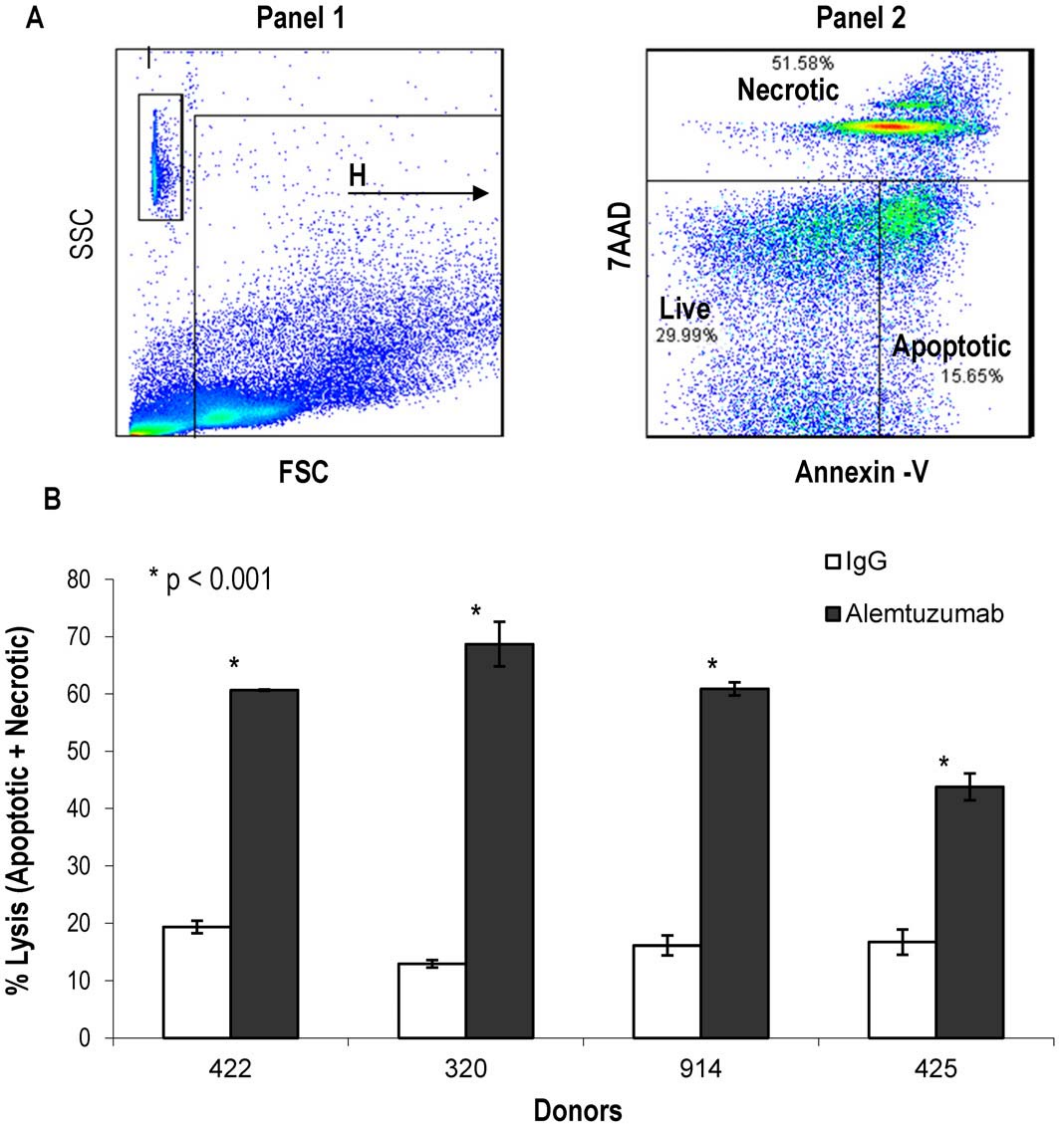
Complement-dependent cytolytic effets of alemtuzumab on human PBMCs. Cells were incubated with 10 µg/ml of alemtuzumab or human IgG1 isotype control in the presence of 10% purified human complement for 3 hrs. Alemtuzumab mediated cytotolysis was assessed by flow cytometry using an LSR-II instrument and a representative dot plot is presented (A). Panel 1 shows the gating strategy. All cells except platelets and counting beads were included into gate H. Panel 2 shows the dot plot analysis of cells from gate H in panel 1. Live cells are negative for Annexin-V and 7AAD. Total lysis was calculated by adding total 7AAD (necrotic) and Annexin V positive (apoptotic) cells. (B). The percentage lysis mediated by alemtuzumab (dark bars) compared to control IgG1(white bars) from each donor is presented. The error bars represent standard deviation (*p<0.05).

We next investigated the extent to which the heterogeneous CD52 levels may confer differences in sensitivity to alemtuzumab mediated cytotolysis. CDC experiments were performed on PBMCs from four donors using a flow cytometry based assay which allows for simultaneous assessment of the total number of dead cells and the absolute number of individual PBMC subsets among cells that survive cytolytic effects. Figure 5A illustrates the flow cytometry strategy used. All lymphocytes and myeloid cells were selected into gate H of Panel 1, based on forward and side scatter, and the counting beads into a separate gate I. The cells outside of gate H which were found to be mainly platelets (data not shown) were excluded from the analysis. As shown in panel 2, based on 7AAD and Annexin V staining, the cells were identified as necrotic (7AAD+ Annexin V+), apoptotic (Annexin V high and 7AAD negative) and live cells (7AAD and Annexin V negative). The sum of necrotic and apoptotic cells constituted the total percentage of dead cells. The individual lymphoid and myeloid subsets within the live cells were identified using the strategy described in figure 1 and 2 and the absolute cell number of each subset was calculated as described in the methods section.

**Figure 6:**
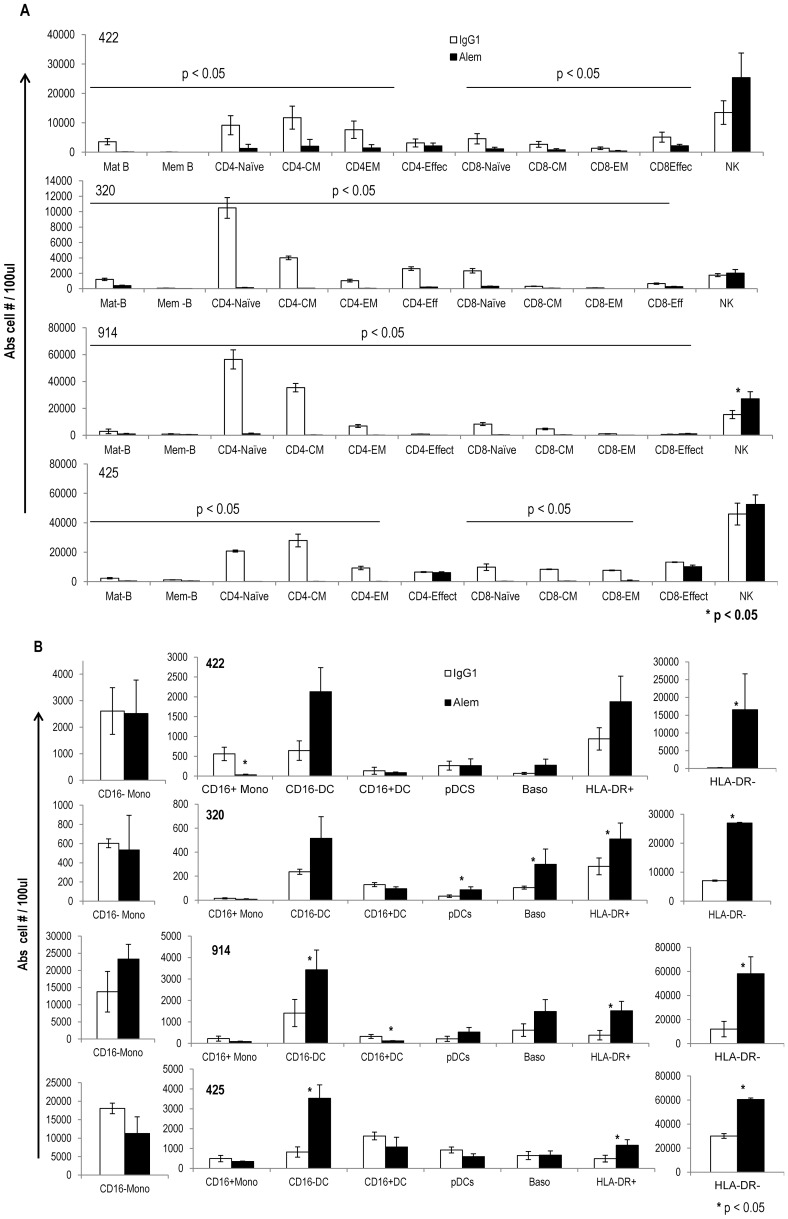
PBMC subsets that survived the cytolytic effects of alemtuzumab. The absolute cell numbers of individual lymphoid (A) and myeloid subsets (B) from PBMCs that survived cytolytic effects (live gate of figure 5) from IgG1 treated (white bars) and alemtuzumab treated (filled bars) of each donor is presented. The error bars represent standard deviation (*p<0.05).

As shown in figure 5B, alemtuzumab mediated signifcant cytolytic effects as compared to control antibody on PBMCs from all four donors (44%–68% with alemtuzumab vs 13–19% with control human IgG1). The absolute number of PBMC subsets that survived the cytolytic effects of alemtuzumab are presented in figure 6A–B. The results show that B and T lymphocyte subsets in alemtuzumab treated samples were significantly reduced compared to human IgG1 treated controls indicating that these cell populations are effectively depleted with alemtuzumab (figure 6A). In contrast, this effect of alemtuzumab was not observed on NK cells and, in fact, there were significantly higher number of NK cells (p<0.05) in the alemtuzumab treated group in one (914) of the the four donors. These results indicate that NK cells are relatively less sensitive than B and T lymphocytes to alemtuzumab mediated cytolytic effects and that NK cells comprise a majority of surviving lymphocytes.

In contrast to lymphocytes, the depleting effects of alemtuzumab on myeloid cells was minimal (Figure 6B). Alemtuzumab did not have significant depleting effects on CD16- monocytes which comprise a majority of the monocyte subset (Fig 1B). Significant depletion (p<0.05) was observed only for CD16+ monocytes from donor 422 and CD16+ DCs from donor 914. CD16+ subsets of monocytes and DCs from donors 320 and 425 survived the CDC effects. CD16− DCs were resistant to alemtuzumab cytolytic effects and in fact were enriched in numbers in all four donors. In the remaining myeloid cell populations, interestingly, there were no significant differences observed between human IgG and alemtuzumab treated groups indicating that alemtuzumab did not exert any significant complement mediated depleting effects on basophils and pDCs. In fact, a significant enrichment of these subsets was frequently observed after alemtuzumab treatment (figure 6B). With the combination of antibodies used in the phenotypic analysis of surviving cells, we could not ascertain the identity of two subsets of myeloid cells presented in figure 6B as HLA-DR + and HLA-DR- populations which are significantly increased (p<0.05) in the alemtuzumab treated group. These have been presented to account for all the cells in the live gate. It is likely that these are contaminating granulocytes with HLA-DR+ cells possibly representing eosinophils and HLA-DR- cells being neutrophils. The apparent increase in the number of cells among myeloid subsets observed in the alemtuzumab treated group is most likely due to a significant loss of other PBMC subsets.

Taken together, the results show that while alemtuzumab exerts complement mediated cytolytic effects on B and T lymphocytes effectively, NK cells, pDCs and basophils are relatively less susceptable, demonstrating a cytolytic effect in proportion to the amount of CD52 on these cells. However, despite expressing CD52 levels comparable to those of T-lymphocytes, CD16+ subsets of monocytes and mDCs were relatively less susceptible to alemtuzumab mediated cytolytic effects suggesting that factors other than antigen density are involved in their resistance to complement mediated killing.

### Monocytes and mDCs Express High Levels of Complement Inhibitory Proteins

**Figure 7:**
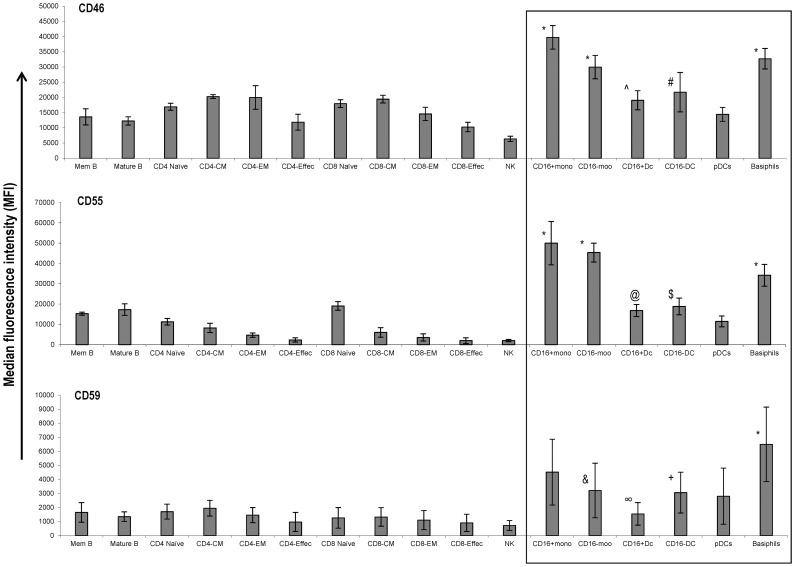
Expression of complement inbitory proteins is higher on myeloid cells than lymphocytes. Expression levels of CD46, CD55 and CD59 proteins were measured by flow cytometry on individual lymphoid and myeloid cell subsets in PBMCs from donors on whom CDC experiments were conducted. Average median fluorescence intensity values of each CIP along with standard deviations are shown. * Significantly higher levels than the lymphocyte subsets (p<0.02) ^Λ^ Significantly higher levels than B cells, effector T and NK cells (p≤0.05) # Significantly higher levels than effector T and NK cells (p≤0.02) @ Significantly higher levels than central memory(CM), effector memory(EM), effector T cells and NK cells (p≤0.02) $ Significantly higher levels than CD4-naïve, central memory(CM), effector meory(EM), effector CD4 and CD8 T cells and NK cells (p≤0.01) & Significantly higher levels than CD8 effector T and NK cells (p≤0.02) ∞ Significantly higher levels than CD8 effector memory and CD4 and CD8 effector T cells (p≤0.03) + Significantly higher levels than effector T cells and CD8-naïve T cell subset (p≤0.05).

Complement inhibitory proteins (CIPs) are widely expressed across many cell lineages and protect cells from complement mediated lysis. There are three major human cell surface CIPs: CD46 (membrane cofactor protein), CD55 (decay acceleration factor) and CD59. Since the myeloid cells were relatively resistant to CDC lysis, we reasoned that differences in the levels of CIP expression between myeloid and lymphoid cells may be responsible for the lack of sensitivity to CDC. Therefore we examined the levels of cell surface expression of CIPs on lymphoid and non lymphoid subsets by flow cytometry. Average relative levels of median fluorescence imtensity (MFI) on mononuclear cell subsets from donors on which CDC experiments were performed are presented in figure 7. All PBMC subsets expressed the three CIPs although average levels of MFI were significantly different. Basophils expressed significantly higher levels (p≤0.02) of all three CIPs compared to all lymphocyte subsets. The CD16+ and CD16− monocytes expressed significantly higher CD46 and CD55 than lymphocytes (p≤0.02). The average CD59 levels on CD16+ monocytes did not differ significantly from that of lymphocyte populations, while CD59 levels on CD16− monocyte were significantly higher compared to only CD8 effector and NK cell subsets (p≤0.02). On CD16+ myeloid DCs, CD46 levels were higher and reached statistical significance (p<0.05) compared to only B cell subsets, effector T cells and NK cells. CD55 expression on the other hand, was significantly higher (p≤0.02) than on all T cell subsets and NK cells but not B cell subsets. The expression level of CD59 was higher on CD16+ mDCs but was not statistically different compared to most of the lymphocyte subsets except effector subsets of CD4 and CD8 T cells (p≤0.03). On CD16− mDCs, the average CD46 expression was significantly higher than for effector T and NK cells (p≤0.02), whereas CD55 was significantly higher than on NK cells and all T cell subsets (p≤0.02) except naïve CD8 T cells. CD59 expression on CD16− mDCs was significantly higher compared to naïve CD8 and effector T cell subsets of both CD4 and CD8 (p≤0.05). Overall, these results show that monocytes and mDCs express higher levels of CIPs than lymphocytes and suggest that these proteins may confer protection to CD16+ monocytes and mDCs from the CDC effects of alemtuzumab.

### Blocking CIPs Partially Reverses the Resistance of Monocytes to the Cytolytic Effects of Alemtuzumab

**Figure 8:**
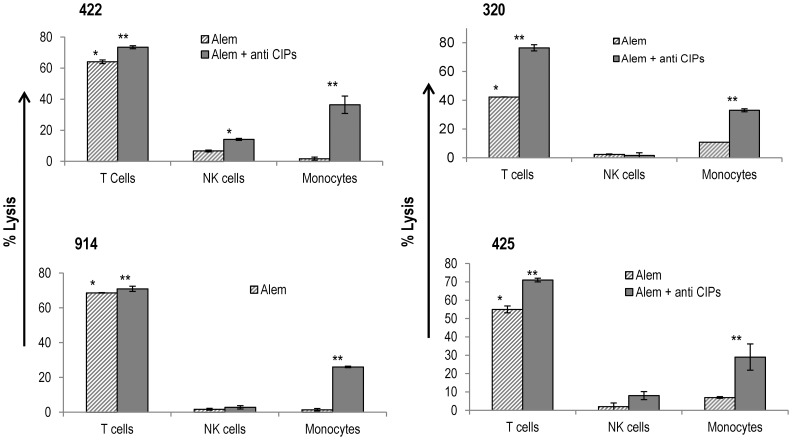
Alemtuzumab mediates robust cytolysis of purified T cells but not NK cells. Blocking anti CIP antibodies partially reverse the resistance of monocytes to lysis by alemtuzumab: PBMCs from each of the 4 donors were sorted using a FACS Aria cell sorter into CD3+ T, CD56+ NK and CD14+ CD11c+ monocytes to >95% purity. Each of these purified populations was subjected to a CDC assay in the presence or absence of 15 µg/ml of anti-CD55 and anti-CD59 antibodies. The percent cytolysis was assessed as described in Fig 5.The hatched bars represent lysis with alemtuzumab without the blocking anti-CD55 and anti-CD59 antibodies and the shaded bars in the presence of blocking antibodies. The control IgG1 values were subtracted before plotting the data. (Background IgG1 range for T cells  = 5–10%; NK cells  = 5−15%; and monocytes  = 15−31%). *p≤0.01 (IgG vs Alem alone) **p≤0.001 (Alem alone vs Alem + anti-CIP abs).

Immune cell subsets are present at widely different ratios within PBMCs. For example, T lymphocytes are present in greater numbers than NK cells or monocytes which are present in lower proportions. To ascertain if the higher cytolytic effect of alemtuzumab on lymphocytes was a direct result of differences in the levels of CD52 rather than due to a relative abundance among PBMCs and to validate the role of CIPs on monocytes, CDC was performed on sorted populations of CD3+ T cells, NK cells and monocytes in the presence or absence of blocking anti-CIP antibodies. As shown in figure 8, alemtuzumab depleted purified T cells to a significant extent with minimal effects on purified NK cells in the absence or presence of anti-CIP antibodies. This confirms that the differential cytolytic effects of alemtuzumab on T lymphocytes and NK cells is primarily due to differences in the levels of CD52. With purified monocytes, alemtuzumab had insignificant lytic effect compared to IgG control in the absence of blocking anti-CIP antibodies. In contrast, the cytolytic activity of alemtuzumab increased significantly (27 -36%) in the presence of antibodies to CD55 and CD59 proteins. However, this cytolytic effect was not to the same extent as observed for CD3+ T cells suggesting that CIPs contribute only partially to the resistance of monocytes to alemtuzumab mediated cytolysis.

## Discussion

In the present study, we sought to systematically assess the qualitative and quantitative levels of CD52 expression on distinct phenotypic PBMC subsets and evaluate the correlation with susceptibility to alemtuzumab mediated complement-dependent cytolysis.

Previous studies have been limited to examination of CD52 expression levels on total B and T lymphocytes. Using radioisotopic methods, it was estimated that lymphocytes express 4.5×10^5^ molecules of CD52 [33]. Ginaldi et. al [14], using a flow cytometric based approach reported CD52 expression as molecules of equivalent soluble fluorochrome values (MESF) with a density of 2−3×10^5^ MESF values on T cells and lower levels ranging from 0.5−2×10^5 ^MESF values on normal B cells. Using a similar approach, Rossman et. al [34], reported higher CD52 levels of 4×10^5^ molecules on B cells in normal controls. Klabusay et.al [27] evaluated CD52 antigen expression and showed that B cells express CD52 at 4×10^5^ MESF units and reported that it translated to 1.9×10^5^ antibody binding capacity units (ABC units). None of these previous studies assessed CD52 expression levels on individual lymphocyte subsets and importantly, the absolute CD52 levels on myeloid cell populations were not investigated. The systematic analysis of PBMCs from normal donors carried out in the present study demonstrates that the qualitative expression of CD52 mirrors quantitative levels and that there is significant differential expression of CD52 among phenotypically distinct subsets of lymphoid and myeloid cells in all donors tested. Importantly, the hierarchical pattern of CD52 expression on PBMC subsets is the same across all donors although the actual numbers of CD52 molecules per cell on any given subset can vary from donor to donor (Table S1). The comprehensive quantitative analysis demonstrates that the number of CD52 binding sites for alemtuzumab is highly variable. The average CD52 levels expressed as ABC units on lymphocytes range from 1.3×10^5^ (SD ±4.3×10^4^) on CD16lo NK cells to 6.3×10^5^ (SD ±6.8×10^4^) on memory B cells while on myeloid cells it ranges from 7×10^4^ (SD ±3×10^4^) on basophils to 4.8×10^5^ (SD ±1.3×10^5^) on CD16+ monocytes in PBMCs from normal donors (Table S1). The CD52 levels on B and T cells reported in the present study are higher than those published from radioisotopic and flow cytometry based studies. Two main reasons could account for these differences: (i) In previous studies, CD52 levels were either assessed on bulk lymphocytes [33] or total B or T cell populations [14], [27], [34], which would provide an average value of high and low CD52 expressing subsets resulting in lower mean numbers of molecules per cell. (ii) Differences in the methodologies used; radioisotopic [33] and MESF based flow cytometry approach [14], [34] as well as a different antibody clone that was used to detect CD52 [27] may also have played a role. MESF is a quantitative value based on the intensity of the fluorescence signal from the sample relative to the signal intensity from a standard fluorochrome coated solution of microbeads and therefore does not provide the actual number of molecules on the cell [35]. ABC units, on the other hand, are derived from the fluorescence intensity of the fluorochrome conjugated antibody binding to a set of beads that are coated with specifically calibrated number of anti-immunoglobulin molecules [35], [36]. We chose the latter strategy and used Quantum Simply Cellular Beads to quantify CD52 antigen density in ABC units as this method represents a relatively more physiological interaction between antigen and antibody.

In the process of evaluating cell surface CD52 expression, we found heterogeneous CD52 expression levels in two PBMC subsets which allowed for further subdivision of these populations. Within CD19+ CD27- naive B cells and pDCs, CD52 expression was variable and each of these subsets could be subdivided into a CD52 hi and CD52 lo cell population (Figure 2C and Table S1). While the heterogeneous expression was consistent and was observed in nineteen of twenty two donors for naïve B cells, it was observed in all the subjects studied for pDCs indicating their frequent presence in PBMCs (Table S1). Whether these subsets are novel populations or represent intermediate stages of differentiation within each of these cell types with characteristic functional features is a subject of future studies. These results, however, demonstrate that CD52 expression represents an additional marker to delineate hitherto uncharacterized subsets among peripheral blood cell populations.

Alemtuzumab mediates cytolytic effects through complement-dependent (CDC) and antibody-dependent cellular cytolytic (ADCC) mechanisms [37]. CDC depends on the aggregation and appropriate juxtaposition of antibody molecules bound to the antigen that results in a conformational change allowing the constant portion of the antibody to fix complement C1q to initiate the complement pathway resulting in lysis of the cell expressing the antigen. This implies that CDC is dependent on the antibody binding capacity and the density of target antigen on the cell. Since CD52 antigen density was variable on PBMC subsets, we investigated whether this variability conferred differences in susceptibility to the CDC effects of alemtuzumab. We found that the cytolytic effect of alemtuzumab was very efficient on B and T lymphocytes that expressed high levels of CD52 (Figure 4B). In contrast, NK cells, basophils and pDCs which express a significantly lower CD52 antigen density were not depleted indicating that cytolytic effects of alemtuzumab were proportional to the amount of CD52 expressed on the cell surface. Interestingly, Lowenstein, et.al [38] observed that in normal peripheral blood, CD4 T cells were more sensitive than CD8 T cells to alemtuzumab mediated CDC and found CD52 expression levels on CD4 T cells to be twice that of CD8 T cells. These data were based on an indirect assay that was used to enumerate dead cells. In the present study, we also found higher CD52 antigen density on CD4 T cell subsets than on CD8 T cell subsets, although this difference was not two fold as reported in the earlier study [38]. Importantly, in contrast to findings of Lowenstein et. al, our data demonstrate that both CD4 and CD8 T cells are equally sensitive to CDC by alemtuzumab. Considering the results of our studies on antigen density and the differential sensitivity to cytolytic effects on PBMC subsets, it is likely that a threshold of CD52 antigen density is required for the CDC activity of alemtuzumab. In this regard, seminal work done by Bindon et. al [33] and others [39], [40] in the late 1980’s has shown the importance of CD52 antigen density on the lytic activity of anti CD52 antibodies including alemtuzumab (Campath). Golay et. al [41] have shown that the success of rituximab in mediating CDC on malignant B cells is highly dependent on CD20 antigen density. A higher level of CDC activity was observed with alemtuzumab compared to rituximab on B-CLL cells and it was found that this difference reflects the amount of target antigens expressed [42]. Therefore, based on the correlation between CD52 antigen density and in-vitro cytolytic effects on PBMC subsets, we speculate that approximately 2.6−2.8×10^5^ ABC units of CD52 antigen density (such as on B & T cells) may be the required lower limit for an efficient complement-dependent cytolysis by alemtuzumab in the absence of other factors that may otherwise influence the cytolytic activity.

In contrast to lymphocytes, monocytes and myeloid dendritic cells were not lysed efficiently with alemtuzumab although these cells express CD52 levels comparable to B and T lymphocytes suggesting that antigen density alone is not sufficient to determine the susceptibility to alemtuzumab mediated lysis. Previous studies also observed relatively lower sensitivity of monocytes and mature monocyte derived blood DCs (moDCs) to alemtuzumab mediated depletion [24], [28], [43]. It was also shown that with anti-CD52 treatment, malignant and normal lymphocytes are depleted, but normal and malignant monocytes are resistant despite expressing large amounts of CD52 antigen [40]. The basis for this resistance, however, was unknown. One potential mechanism could be the expression of complement inhibitory proteins (CIPs) whose function in protecting from autologous complement mediated lysis has been well documented [44], [45]. Our studies indicate that compared to lymphocytes, monocytes and mDCs express significantly higher levels of CIPs. Additional experiments conducted in the presence of neutralizing anti-CD55 and anti-CD59 antibodies on purified population of monocytes did reveal a significant increase in their susceptibility to alemtuzumab mediated cytolysis. However, the average increase in lysis was 31% above the background which is still lower than the extent to which T cells were depleted by alemtuzumab treatment. These data indicate that the higher levels of CIPs on monocytes contribute only partially to their resistance to alemtuzumab mediated lysis. Thus it is likely that apart from complement regulatory proteins, there may be additional cell intrinsic factors that are contributing to the relative resistance of high CD52 expressing myeloid cells to cytolysis by alemtuzumab.

In summary, our results demonstrate that human peripheral blood mononuclear cells exhibit significant variability in quantitative CD52 expression levels and show differential sensitivity to alemtuzumab mediated complement-dependent cytolysis in vitro. Our findings show that B and T lymphocytes, which express high CD52 antigen density, are most susceptible while NK cells, pDCs and basophils which express low CD52 antigen density are least susceptible to lysis demonstrating a direct correlation between antigen density and the complement-dependent cytolytic effects of alemtuzumab on these cells. However, monocytes and mDCs were not susceptible to lysis despite the presence of high CD52 antigen density. While expression of high levels of CIPs is one contributing factor, there are most likely additional unknown cell intrinsic factors that seem to confer resistance to these cells. Overall, alemtuzumab mediated CDC results in efficient lysis of cells from the adaptive immune system while leaving the components of the innate immune system relatively intact. These results further our understanding of the mechanism of action of alemtuzumab and its potential therapeutic benefit in autoimmune disease indications.

## Supporting Information

Table S1 **CD52 antigen density on individual PBMC subsets from each donor.**
(DOCX)
