# Supplementary material for: Human Peripheral Blood Mononuclear Cells Exhibit Heterogeneous CD52 Expression Levels and Show Differential Sensitivity to Alemtuzumab Mediated Cytolysis
Source: PLoS One. 2012 Jun 25;7(6):e39416. doi: 10.1371/journal.pone.0039416 (PMC3382607; doi:10.1371/journal.pone.0039416)
Supplement: Table S1 — CD52 antigen density on individual PBMC subsets from each donor. (DOCX) [file pone.0039416.s001.docx]

Table S1.

Alemtuzumab binding capacity (ABC units) to CD52 antigen on human PBMC subsets

| **Donor** | **831** | **914** | **320** | **3165** | **422** | **816** | **425** | **720** | **917** | **1013** | **1128** |
| --- | --- | --- | --- | --- | --- | --- | --- | --- | --- | --- | --- |
| Mem. B | 641,347 | 668,344 | 627,596 | 603,383 | 547,431 | 571609 | 666597 | 658152 | 693220 | 664748 | 634413 |
| Naïve B CD52hi | 463,416 | 481,310 | 509,765 | 438,218 | 456,442 | 417649 | 532080 | 518064 | 520092 | 525291 | 493361 |
| Naïve B CD52lo | ND | ND | ND | 24853 | 17177 | 17738 | 91444 | 60301 | 55919 | 66424 | 66315 |
| CD4 Naïve | 593253 | 567330 | 619964 | 558945 | 548006 | 529940 | 655890 | 599498 | 580829 | 637713 | 630268 |
| CD4-CM | 571258 | 537408 | 452901 | 550588 | 387661 | 509836 | 585647 | 380245 | 563096 | 419298 | 553172 |
| CD4-EM | 474175 | 414987 | 348984 | 323408 | 226417 | 243560 | 357671 | 352529 | 432584 | 349220 | 374226 |
| CD4-Effector | 494746 | 505552 | 534092 | 459416 | 454970 | 289106 | 560527 | 428043 | 484704 | 532080 | 446535 |
| CD8-Naïve | 460718 | 395297 | 509640 | 346397 | 421217 | 316758 | 454831 | 376808 | 419761 | 462423 | 393993 |
| CD8-CM | 372635 | 356034 | 333267 | 315749 | 314253 | 288674 | 379259 | 328860 | 367927 | 360362 | 280619 |
| CD8-EM | 192106 | 195654 | 283022 | 278122 | 259482 | 224514 | 231990 | 325877 | 331420 | 356531 | 278471 |
| CD8-Effector | 186266 | 164717 | 130580 | 177326 | 182638 | 145263 | 195228 | 185910 | 179864 | 231602 | 176978 |
| CD16- NK | 130040 | 123970 | 85674 | 174771 | 193419 | 154375 | 142901 | 213912 | 145873 | 228438 | 198211 |
| CD16+ NK | 130040 | 123970 | 85674 | 174771 | 193419 | 154375 | 142901 | 213912 | 145873 | 228438 | 198211 |
|  |  |  |  |  |  |  |  |  |  |  |  |
| CD16-mono | 335936 | 113306 | 226760 | 378322 | 252444 | 337789 | 308592 | 282877 | 312743 | 404002 | 336635 |
| CD16+ mono | 415213 | 217298 | 334148 | 606358 | 511313 | 538264 | 549196 | 401162 | 490782 | 598458 | 559557 |
| CD16- DCs | 199542 | 240770 | 135524 | 268910 | 141890 | 173020 | 290924 | 251293 | 473010 | 599112 | 384738 |
| CD16+ DCs | 558630 | 546861 | 462277 | 609433 | 545071 | 598458 | 452559 | 376023 | 482515 | 565251 | 512119 |
| CD52hi pDCs | 187184 | 48727 | 76105 | 126923 | 89076 | 144947 | 284324 | 244520 | 471075 | 594547 | 401162 |
| CD52lo pDCs | 75609 | 22896 | 26919 | 6217 | 9399 | 17011 | 81975 | 76689 | 64832 | 165529 | 75609 |
| Basophils | 74135 | 102227 | 47778 | 52372 | 30390 | 49833 | 118399 | 142853 | 74659 | 61626 | 20428 |

ND = Not detected, CM= central memory, EM=Effector memory, NK=Natural killer, DC=Dendritic cell, mono = Monocytes, pDC = Plasmacytoid dendritic cell

Table S1 cont--

| **Donor** | **G115** | **G22** | **G81** | **G117** | **G120** | **G23** | **G26** | **G28** | **G54** | **G90** | **G102** | **Avg** | **STD** |
| --- | --- | --- | --- | --- | --- | --- | --- | --- | --- | --- | --- | --- | --- |
| Mem. B | 599263 | 765355 | 778970 | 668003 | 688120 | 555351 | 668665 | 506236 | 612886 | 619704 | 523832 | **634692** | **68919** |
| Naïve B CD52hi | 417179 | 463968 | 554744 | 506341 | 440363 | 302632 | 359699 | 306176 | 378438 | 307565 | 298562 | **440516** | **82295** |
| Naïve B CD52lo | 27562 | 148678 | 132825 | 120371 | 120371 | 75936 | 59318 | 75511 | 106856 | 75298 | 80758 | **74929** | **38421** |
| CD4 Naïve | 551296 | 728263 | 761937 | 793826 | 673289 | 552210 | 554035 | 443229 | 513031 | 512718 | 449597 | **593412** | **90092** |
| CD4-CM | 580254 | 520829 | 716327 | 778702 | 697352 | 522383 | 580851 | 491380 | 557071 | 577861 | 515635 | **547716** | **96572** |
| CD4-EM | 443229 | 605059 | 615329 | 608586 | 385071 | 432168 | 476676 | 383330 | 477319 | 214132 | 303266 | **401906** | **113002** |
| CD4-Effector | 157505 | 520829 | 572865 | 449816 | 360532 | 361601 | 455388 | 240661 | 355407 | 195054 | 224421 | **412902** | **123192** |
| CD8-Naïve | 485114 | 715313 | 712916 | 751379 | 677995 | 526313 | 491909 | 487772 | 486710 | 472167 | 441797 | **491238** | **120045** |
| CD8-CM | 279068 | 381469 | 438485 | 471952 | 321199 | 230215 | 261201 | 271445 | 315847 | 340348 | 217011 | **328449** | **61826** |
| CD8-EM | 257708 | 351215 | 355765 | 343258 | 279852 | 226411 | 237753 | 230074 | 262272 | 229934 | 200552 | **269636** | **53672** |
| CD8-Effector | 157505 | 292931 | 321323 | 325408 | 238585 | 192959 | 185709 | 178044 | 233017 | 217728 | 222725 | **205559** | **51904** |
| CD16- NK | 101005 | 108347 | 129188 | 129188 | 143690 | 69029 | 117678 | 95609 | 113688 | 100049 | 80135 | **135418** | **43632** |
| CD16+ NK | 71648 | 168522 | 232318 | 247942 | 174943 | 88901 | 177426 | 172135 | 193858 | 188894 | 86892 | **163412** | **49891** |
|  |  |  |  |  |  |  |  |  |  |  |  |  |  |
| CD16-mono | 217154 | 196993 | 277628 | 185253 | 130578 | 309580 | 179433 | 202471 | 198775 | 203501 | 243968 | **256125** | **78180** |
| CD16+ mono | 296774 | 414586 | 703111 | 292803 | 348209 | 343157 | 467643 | 600542 | 702369 | 556262 | 636628 | **481083** | **137931** |
| CD16- DCs | 204382 | 162809 | 150656 | 124808 | 142843 | 215861 | 204823 | 181585 | 116414 | 248488 | 233995 | **233882** | **117945** |
| CD16+ DCs | 312719 | 453315 | 492333 | 324430 | 173542 | 250124 | 547739 | 253585 | 278414 | 462235 | 290616 | **434011** | **129432** |
| CD52hi pDCs | 67041 | 218301 | 208186 | 167439 | 111857 | 209933 | 137278 | 132308 | 199368 | 124279 | 118758 | **198334** | **135502** |
| CD52lo pDCs | 18672 | 57447 | 56739 | 56976 | 24305 | 11799 | 39369 | 36151 | 37676 | 30265 | 27345 | **46338** | **35757** |
| Basophils | 68369 | 67041 | 133686 | 78465 | 82410 | 72297 | 70779 | 54117 | 81792 | 48972 | 58151 | **72308** | **30230** |

CM= central memory, EM=Effector memory, NK=Natural killer, DC=Dendritic cell, mono=Monocytes, pDC = Plasmacytoid dendritic cell, Avg= Average, STD=Standard deviation
